# Insights from mathematical modelling and quantitative analysis on the proposed WHO 2030 targets for visceral leishmaniasis on the Indian subcontinent

**DOI:** 10.12688/gatesopenres.13073.1

**Published:** 2019-10-29

**Authors:** NTD Modelling Consortium Visceral Leishmaniasis Group

**Keywords:** Visceral leishmaniasis, Modelling, WHO guidelines, Elimination as a Public Health Problem, Control, Feasibility, NTD Modelling Consortium

## Abstract

Visceral leishmaniasis (VL) is a neglected tropical disease (NTD) caused by *Leishmania *protozoa that are transmitted by female sand flies. On the Indian subcontinent (ISC), VL is targeted by the World Health Organization (WHO) for elimination as a public health problem by 2020, which is defined as <1 VL case (new and relapse) per 10,000 population at district level in Nepal and sub-district level in Bangladesh and India. WHO is currently in the process of formulating 2030 targets, asking whether to maintain the 2020 target or to modify it, while adding a target of zero mortality among detected cases. The NTD Modelling Consortium has developed various mathematical VL transmission models to gain insight into the transmission dynamics of VL, identify the main knowledge gaps, and predict the feasibility of achieving and sustaining the targets by simulating the impact of varying intervention strategies. According to the models, the current target is feasible at the appropriate district/sub-district level in settings with medium VL endemicities (up to 5 reported VL cases per 10,000 population per year) prior to the start of the interventions. However, in settings with higher pre-control endemicities, additional efforts may be required. We also highlight the risk that those with post-kala-azar dermal leishmaniasis (PKDL) may pose to reaching and sustaining the VL targets, and therefore advocate adding control of PKDL cases to the new 2030 targets. Spatial analyses revealed that local hotspots with high VL incidence remain. We warn that the current target provides a perverse incentive to not detect/report cases as the target is approached, posing a risk for truly achieving elimination as a public health problem although this is taken into consideration by the WHO procedures for validation. Ongoing modelling work focuses on the risk of recrudescence when interventions are relaxed after the elimination target has been achieved.

## Disclaimer

The opinions expressed herein are those of the authors and do not necessarily reflect the views of the World Health Organization. Publication in Gates Open Research does not imply endorsement by the Gates Foundation.

## Background

Visceral leishmaniasis (VL) is a neglected tropical disease (NTD) caused by *Leishmania* protozoa that are transmitted by infected female sand flies. Most individuals remain asymptomatic, but those that develop symptoms experience long-lasting fever and hepatosplenomegaly, and die when left untreated ^1^. About 5–10% of treated individuals develop a skin condition called post-kala-azar dermal leishmaniasis (PKDL) ^2^. Interventions include active case detection (ACD) of VL followed by prompt treatment, and vector control through indoor residual spraying of insecticide (IRS).

The World Health Organization (WHO) has targeted the elimination of VL as a public health problem on the Indian subcontinent by 2020, defined as <1 VL case (new case and relapse) per 10,000 population per year, at the district level in Nepal and sub-district level in Bangladesh and India ^3^. To achieve this target, the associated WHO guidelines describe four phases: a pre-control preparatory phase; a 5-year attack phase designed to bring the incidence below 1 per 10,000 per year; a consolidation phase where incidence is kept below the target for 3 years; and a maintenance phase to ensure sustainable reductions in incidence ^4^. The interventions associated with these phases entail different levels of ACD and IRS. With 2020 approaching, WHO is in the process of formulating targets for 2030 for the Indian subcontinent, asking stakeholders in the VL community whether to maintain the 2020 target or to modify it, while adding the target of zero mortality among detected cases. The stakeholders include endemic country representatives, implementing partners, donors, mathematical modellers, WHO staff, and others, and were all invited to provide ideas and feedback on the new 2030 WHO targets (April–July 2019).

Mathematical transmission models have proven useful tools in gaining insight into the transmission dynamics of infectious diseases, highlighting knowledge gaps, and predicting the impact of interventions, to ultimately inform policy makers in formulating optimal strategies ^5^. The Bill-and-Melinda-Gates-Foundation-funded NTD Modelling Consortium was founded in 2014, bringing together multiple groups working on the modelling of NTDs, including VL, with VL modelling teams from The Netherlands (Erasmus MC) and the United Kingdom (Warwick University, London School of Tropical Medicine and Hygiene, and Oxford University) ^6^. In close collaboration with disease experts and policy makers, the VL teams captured the transmission dynamics of VL with mathematical models ^7, 8^, compared their models through geographical cross-validation ^9^, and published a joint policy paper ^10^. Even though the models had certain different underlying assumptions regarding the main knowledge gaps, such as the role of asymptomatic individuals, PKDL cases, the duration of immunity, and the actual quality of the interventions, the models generally agreed on the predicted trends towards elimination. In this Open Letter we present an overview of the main VL modelling outcomes to support the development of the new WHO 2030 VL targets.

## Insights gained from mathematical modelling analyses

Model predictions have suggested that the current intervention guidelines recommended by the WHO are sufficient to reach the elimination target in areas with moderate VL incidence (up to 5 reported VL cases per 10,000 population per year) prior to the start of interventions. However, additional interventions, such as extending the WHO attack phase (intensive IRS and ACD), may be required to achieve the elimination target in regions with high pre-control incidence, depending on the relative infectiousness of different disease stages. As VL incidence decreases, the pool of susceptible individuals will grow, creating the potential for new outbreaks ^10^.

Despite the overall decrease in VL incidence at sub-district level, clusters or ‘hotspots’ of high incidence within sub-districts remain. We studied these hotspots at hamlet (sub-village) level using geospatial analyses and found significant spatial and temporal heterogeneity in VL incidence. Based on these findings, we argue for considering lower geographical units for evaluation of control after the targets at district/sub-district level have been achieved ^11^. Another key issue is the spatial spread of infection at an even smaller scale, between human hosts. Our modelling work suggests that case-to-case transmission is local (≤500 m) ^11– 13^, which would support the current range of IRS around affected villages. However, the value of IRS, as it is currently implemented, has been questioned ^14, 15^, whereas there is some indirect evidence for the impact of diagnosis and treatment ^14, 16, 17^, making case detection pivotal in the elimination programme. Moreover, even though this is currently not the target, no geographical areas have been “cleared” of transmission, suggesting that current tools are not sufficient to achieve zero transmission and may have to be implemented indefinitely to maintain control. Incomplete understanding of the spatiotemporal variation in VL incidence and its importance for achieving elimination as a public health problem and/or interrupting transmission undermines our ability to provide advice on the most appropriate geographical level to evaluate programme impact.

Recent xenodiagnostic studies have found PKDL can be at least as infectious as VL, depending on skin lesion type and severity ^18^. When incorporating this into our models, PKDL becomes an important source of transmission, especially when nearing the low-incidence VL elimination target ^10, 19^, likely challenging the maintenance of the elimination target on the Indian subcontinent. When adding a hypothetical PKDL control strategy (e.g. preventing 95% of PKDL) to the existing WHO strategy, the elimination target can be achieved as much as 8 years earlier, when keeping the assumption that only 2.5% of past VL cases develop PKDL constant ^19^. Currently, the main intervention programmes do not include PKDL control. The main reason is that the options for diagnosing and treating this skin condition are limited. Ideally, active PKDL case-finding and treatment would be implemented, through long-term follow-up of VL patients, patient education to report themselves to the health clinic when symptoms of skin discoloration or rash appear, and educating community workers on recognizing PKDL. However, the suggested strategies are operationally difficult and require the availability of safe and effective treatment. There is a clear need for better VL treatment options, including a vaccine or immunomodulator to avert any PKDL development, and diagnostics for PKDL.

It is unclear to what extent interventions have contributed to the decline in VL incidence in addition to the secular trend in VL incidence, as some decline may be due to long-term (~15-year) natural cycles ^20– 22^. However, transmission models suggest that interventions are likely to have contributed significantly to the decline over the last 5 years. Large village-level outbreaks of disease are still to be expected and have been observed in the field (e.g. Kosra ^23^) ^24, 25^, with recent stochastic modelling work reproducing those trends ^12^. From the point of view of achieving the target of 1 per 10,000 cases in each block (sub-district level in India), due to the high variability in case numbers at this level, it is statistically implausible that the target can be achieved in all blocks simultaneously. This variation means that whilst low endemicity is maintained, the current target will fail at some point, as has occurred recently in Nepal ^26^. This suggests that the choice of administration level at which to assess elimination as a public health problem is critical to attaining and maintaining the target.

Table 1 summarizes the modelling insights and challenges for reaching the WHO 2030 targets. In Table 2 we summarize the priority questions that were identified in discussions with WHO and present how modelling can address this.

**Table 1.** Summary of modelling insights and challenges for reaching the WHO 2030 targets for visceral leishmaniasis.

Current WHO 2020 target on the Indian subcontinent:	Elimination of VL as a public health problem, defined as <1 VL case (new and relapse) per 10,000 population per year, at district/sub-district level.
Proposed WHO 2030 target on the Indian subcontinent:	Elimination of VL as a public health problem, defined as <1 VL case (new and relapse) per 10,000 population per year, at district/sub-district level and zero deaths among detected cases. The administrative level could be adjusted if discussion between VL stakeholders, WHO, and ministries of health (MOH) of affected countries lead to acceptance by the MOH to modify it. Reducing the administrative level, would lower the population size and therefore better reflect the population at risk of VL, but would enhance the variability in incidence.
Is the new target technically feasible under the current disease strategy?	VL incidence and relapse of <1/10,000/year seems feasible in most regions at district level and higher, but not at smaller geographical scales.
If not, what is required to achieve the target? (updated strategy, use of new tools, etc.)	Additional data streams (to ensure case detection is complete); means to assess and prevent occult transmission; means to predict outbreaks; means to detect and treat PKDL more completely and earlier; more specific diagnostics for earlier treatment of cases, and the reporting of relapses.
Are current tools able to reliably measure the target?	No, the targets are defined in terms of numbers of reported diagnoses and deaths, which likely provides an incomplete picture of actual case incidence and mortality, and poses a perverse incentive for complete reporting.
What are the biggest unknowns?	The duration of immunity, the impact of indoor residual spraying of insecticide (IRS), the role of asymptomatic individuals, true VL incidence (besides reported incidence), and how changes in detection delay and population coverage affect VL transmission.
What are the biggest risks?	Presence of PKDL and lack of tools to effectively diagnose, treat, and prevent. The current target provides a perverse incentive to not detect/report cases as the target is approached. Risk of recrudescence when interventions are relaxed after achieving the low-incidence target. Continued outbreaks at village level combined with increasing susceptibility leading to widespread and sustained epidemics.

## Practical implications of the currently proposed targets

### Timeline and feasibility

The VL elimination target of <1 VL case per 10,000 population per year is feasible in most sub-districts (and larger administrative units) ^7, 9, 10^, but at lower geographical levels small outbreaks will continue to occur with case incidence above the target due to ongoing transmission from undiagnosed VL cases, PKDL cases, potentially asymptomatic cases, and those with HIV-VL co-infection. The goal of zero deaths among detected cases is feasible with current treatments if there is stringent post-treatment follow up. However, undiagnosed cases may still die of VL as they remain untreated.

Moreover, operationally it is challenging to successfully implement active case detection in all villages; the best intervention would prevent outbreaks, and not “follow” them. Currently, it is not possible to predict when and where outbreaks will occur in order to target control efforts. Future modelling work may be able to help identify useful markers to predict high-risk areas.

### Ability to sustain achievement of the goal

Continued case detection at the current level is required otherwise there is a risk that incidence will resurge ^10, 19^. Active follow-up of treated cases is required to ensure treatment success and detect relapse cases and cases of PKDL (90% of PKDL cases occur among treated VL cases) ^2^. Treatment availability and effectiveness will need to be sustained. New drugs will eventually need to be developed, ideally those that do not require a cold chain. Health system and community awareness will need to be maintained to ensure cases are detected. A more specific diagnostic that could detect pre-clinical cases without delay could have major impact ^17^.

### Measuring the target

The numbers of cases diagnosed at the block level is very heterogeneous (many zeros and some occasional high numbers, e.g. 36 in one block in one month in India). With >600 endemic blocks in India it is statistically infeasible that all blocks will be below target for 3 consecutive years. This “failure” is in spite of the undoubted reduction in number of cases. Moreover, the current target creates a perverse incentive to not detect/register cases when approaching, and after having reached, the target (see paragraph below “Where are the places of perverse incentive?”). Models suggest complete case detection and treatment may be more important than reducing the delay to treatment to <45 days among diagnosed cases (while still missing cases) to reduce transmission ^17^. Nevertheless, the current target ensures programme effectiveness and appropriate health system engagement. Xenomonitoring, antigen detection, and serosurveillance are potential independent measures of transmission that are being developed under the SPEAK India consortium. Measuring mortality can be challenging, as it requires governments to follow the TDR/WHO manual for death definition and share these data with WHO as part of the validation procedure.

### Considerations of cost

Lenk *et al.* estimated how much of the income loss faced by individuals affected with VL would be avoided by reaching the global WHO targets, based on the number of averted cases and deaths when achieving and sustain#ing the targets ^27^. The total economic benefit from globally averted productivity loss in the period 2011-2030 when reaching the WHO targets for VL was calculated to be 21.3 billion I$ ^1^, of which 15.1 I$ (70%) are from reaching the targets in India. The out-of-pocket payments (OPPs) for an individual with VL in India were found to be I$ 354.75 ^28^. According to the literature, 80% of all patients were being treated in India ^29– 31^, and this percentage was assumed to remain constant until 2030 in this study. The percentage of patients paying for treatment was assumed to linearly decrease to zero in 2030, considering universal health coverage. The total economic benefit from globally averted OPPs for VL added up to 270 million I$ for the period 2011-2030.

WHO has estimated that the economic benefits of achieving the VL target are higher than the cost of the required investments, resulting in a net return on investment ^32^. It was calculated that for VL, the net benefit ^2^ was 3.5 USD for every dollar invested in the period 1990-2030 (adapted from 32). Hence, it was considered that the financial efforts that are required to reach the WHO VL targets are justified.

## Where are the risks that need to be mitigated to achieve the stated goals?

The current focus of the control programme is in the so-called “endemic” administrative units, although the actual definition used for this classification is unclear. This also means that the “non-endemic” units can have unobserved and uncontrolled transmission. The experience of the control programme has been that the “endemic” region appears to have expanded even as the incidence of cases has fallen ^25^ possibly due to human mobility combined with late development of PKDL and potential transmission from asymptomatic individuals, and/or an increase in case detection. Moreover, human mobility and migration might provide a constant “trickle” of transmission. Infrequent large epidemics (e.g. Kosra ^23^), and continued low-level transmission in Nepal ^26, 34^ suggest that both of these occur.

Overall, poor understanding of the spatiotemporal variation in VL incidence and its importance for achieving control and/or interrupting transmission undermines our ability to provide advice on the most appropriate geographical level to evaluate programme impact. Fine-scale geographical measurement will lead to high variation over time and space whereas a lower spatial resolution (i.e. aggregating M&E results over larger areas) will come with a risk of missing programme failure at a more local level. Additional risks include the perverse incentive to not detect/register cases in order to ensure the target is achieved; natural disasters that could disrupt detection of cases (e.g. flooding in Raghopur block, India in August 2017 disrupted control); and the potential rise of HIV and its interaction with VL that could drive local resurgent epidemics ^35^.

## Where are the places of perverse incentive?

Diagnosis and treatment are critical for ongoing VL control (i.e. to attain and maintain elimination as a public health problem). Targets should encourage diagnosis and treatment, but the “knife-edge” target encourages cases not to be diagnosed/treated/reported if it will mean that the block (and therefore country) fails to reach or sustain the target. The WHO validation process includes analyses to ensure that the claimed elimination is not due to underreporting ^4^. The average block size in India is ~200,000, at which the target is 20 cases: 21 is failure and 19 is success. At the recent meeting WHO suggested to create other categories instead of success/fail such as very low/low/medium/high/very high. Such a target could encourage case finding and detach the success/failure of the target from finding of a single case in a block. Another suggestion is to report a detected case as a person who has been diagnosed, treated, and has not died. In this way it could be a positive incentive by saying “number of lives saved”. Other suggestions include setting an incidence target of 1/20,000 at the district level, or setting a target to increase the proportion of cases coming from low-incidence blocks, such as done in the Americas.

## Priority questions

**Table 2.** Summary of priority questions identified in discussion with WHO and how modelling can address this.

Priority questions identified in discussion with WHO	How can modelling address this?
What would be an ambitious but achievable goal for 2030?	To switch the target to <1/20,000/year at the district level or making sure that the vast majority of cases is coming from blocks with incidence <1/20,000/year in the previous year.
How good can the health system be? What would be a realistic number of deaths seen in treated individuals? (in the context of zero deaths goal)	Our current models do not specifically tackle how the health system can change to reduce mortality in treated cases; what we can say is how much mortality in the population as a whole can be reduced if more undetected cases are found and treated.
Once the targets are met, would it lead naturally to the elimination of transmission of VL?	We have attempted to answer this question in existing modelling publications, notably ^6^. An important factor is the presence of a large susceptible population once the targets are met, potentially with PKDL cases still present that remain infectious for years, that could cause recrudescence of infection. The existing models now need to be reviewed and revised to reflect improved understanding of local transmission in small populations that are interlinked.
What do the models suggest is the likely probability of achieving the goals?	According to the deterministic transmission models, the 2020 target is feasible at the appropriate district/sub-district level in settings with medium VL endemicities (up to 5 reported VL cases per 10,000 population per year) prior to the start of the interventions. However, in settings with higher pre-control endemicities, additional efforts may be required. Initial results from statistical forecasting also suggest that the 2020 elimination target is unlikely to be met everywhere, despite the success of the programme in reducing the incidence of cases.
How would improvements in the programme (such as decreasing time to diagnosis and improving efficacy of IRS) reduce the timelines to achieve the target.	Increasing the duration of the WHO attack phase from 5 (current guidelines) to 10 years in high pre-control endemicity settings (10/10,000/year) was simulated to bring the elimination target forward by at least 5 years ^10^. Currently we are investigating how changes in detection delay and population coverage affect VL transmission and the timeline to achieving the target.
How can improvements of the programme to achieve a better case detection impact meeting the target of number of cases per block?	As we noted above, the current target offers perverse incentives, as if case detection is poor, the target is more likely to be met, even though the region would then likely not meet the WHO validation of elimination criteria. This can potentially lead to underdiagnosis/ underreporting and large future outbreaks. Better data on the detection process would enable more detailed modelling. We are currently considering the impact of variation in detection success.

## Data availability

No data are associated with this article.

## Footnotes

^1^An international dollar (I$) would buy in the cited country or countries a comparable amount of goods and services a U.S. dollar would buy in the United States ^33^.

^2^Only investments in individual management of VL were included, as well as active case finding and vector control (only in areas of the Indian subcontinent that are not co-endemic with malaria).
