# Supplementary material for: Insights from mathematical modelling and quantitative analysis on the proposed WHO 2030 targets for visceral leishmaniasis on the Indian subcontinent
Source: Gates Open Res. 2019 Oct 29;3:1651. [Version 1] doi: 10.12688/gatesopenres.13073.1 (PMC7416083; doi:10.12688/gatesopenres.13073.1)
Supplement: Peer review history

# Peer review: Reviewer response for version 1

**DOI:** 10.21956/gatesopenres.14209.r29193

**Published:** 2020-08-17

**Authors:** Patricia Sampaio Tavares Veras

**Affiliations:** 1 Laboratory of Host-Parasite Interaction and Epidemiology, Gonçalo Moniz Institute, Fiocruz-Bahia, Salvador, Brazil

In this Open Letter, the "NTD Modelling Consortium" analyses the mathematical transmission models of VL they developed with a variety of purposes: to understand transmission dynamics and identify the main knowledge gaps of the disease and to evaluate for 2030 which possible interventions could impact on disease control in the Indian continent. Using these analyses, they found that PKDL may pose to reaching and sustaining the VL targets since it becomes an essential source of transmission. Therefore, they advocate adding as principal intervention control of PKDL cases to achieve the new 2030 targets. Spatial analyses also revealed that local hotspots with high VL incidence would remain, despite the main target of reducing VL cases could be achieved.

 This referee agrees that this Letter seems well elaborated and, in general, it clearly describes the background, aims, and the mathematical models used that led them to propose the new targets for VL in 2030. However, once the authors properly addressed the following points, manuscript clarity would be improved. Please include a description of their hypothesis and conclusions in the Abstract section. The “Considerations of cost” subsection seems unnecessary for the main objective of this Letter. This referee also suggests that the authors include a definition of “Perverse incentive” for clarity. Finally, the manuscript would gain in clarity if they include a “Conclusion section” at the end of the document.

Does the article adequately reference differing views and opinions?

Yes

Are all factual statements correct, and are statements and arguments made adequately supported by citations?

Yes

Is the Open Letter written in accessible language?

Yes

Where applicable, are recommendations and next steps explained clearly for others to follow?

Yes

Is the rationale for the Open Letter provided in sufficient detail?

Partly

Reviewer Expertise:

Parasitology, Treatment strategy for cutaneous leishmaniasis, Cell Biology of Host-Leishmania interaction

I confirm that I have read this submission and believe that I have an appropriate level of expertise to confirm that it is of an acceptable scientific standard, however I have significant reservations, as outlined above.

# Peer review: Reviewer response for version 1

**DOI:** 10.21956/gatesopenres.14209.r29187

**Published:** 2020-08-17

**Authors:** Hira L Nakhasi

**Affiliations:** 1 Division of Emerging and Transfusion Transmitted Diseases, Center for Biologics Evaluation and Research, Food and Drug Administration, Silver Spring, MD, USA

The authors have very well laid out the current status of Leishmaniasis in the Indian subcontinent. They have used mathematical modelling with various assumptions to achieve WHO target of elimination of Leishmaniasis. They have raised important flaws in the approaches such as active case detection (ACD)and indoor residual spraying (IRS) that have been used for the elimination i.e. incidence of below 1:10,000 per year and the reason that such target could not be achieved by 2020. They also have proposed a new target date of 1:20,000 to achieve elimination by 2030. Among the various hurdles in achieving the elimination of Leishmaniasis as pointed out by the authors is the spread of the disease in non-endemic areas and the phenotype of the *L donovani* from visceral to cutaneous form in some regions of the subcontinent as well as in India.

 One of the areas which the modelers should have considered besides ACD, IRS, drug treatment and other incentives, the use of vaccines for the elimination program. They should use in their future assumptions vaccines that are cross protective against various species and against visceral, cutaneous and post kala azar dermal leishmaniasis.

 In the opinion of this reviewer greater success in the elimination of Leishmaniasis can be achieved by having a safe and effective Leishmania vaccine besides the existing interventions.

Does the article adequately reference differing views and opinions?

Yes

Are all factual statements correct, and are statements and arguments made adequately supported by citations?

Yes

Is the Open Letter written in accessible language?

Yes

Where applicable, are recommendations and next steps explained clearly for others to follow?

Yes

Is the rationale for the Open Letter provided in sufficient detail?

Yes

Reviewer Expertise:

Leishmania pathogenesis and vaccine immunology

I confirm that I have read this submission and believe that I have an appropriate level of expertise to confirm that it is of an acceptable scientific standard.

# Peer review: Reviewer response for version 1

**DOI:** 10.21956/gatesopenres.14209.r29188

**Published:** 2020-08-07

**Authors:** Megha Raj Banjara

**Affiliations:** 1 Central Department of Microbiology, Tribhuvan University, Kirtipur, Kathmandu, Nepal

The authors described through mathematical modelling the feasibility of visceral leishmaniasis (VL) elimination in Indian sub-continent by 2030 based on the current target for elimination, which has been defined as VL incidence <1 per 10,000 population at district/sub-district level. The manuscript identified the knowledge gaps and impact of VL control interventions. There are limitations of the models- how many predictors have been kept in the model and how have those variables been prioritized in the model. For example, the authors had assumptions on certain important drivers of transmission dynamics. Nepal has achieved elimination target in 2013 in previously VL endemic districts which have VL incidence 18 times more in 2006 than the elimination target; however, authors mentioned that the current target is feasible up to 5 reported VL cases per 10,000 population per year prior to the start of the interventions. Therefore, I suspect the model with roles of interventions in VL elimination.

1. I suggest to provide details of model and predictors included in the model.
2. The authors mentioned that "VL is targeted by the World Health Organization (WHO) for elimination as a public health problem by 2020" but I think it is the target of respective countries and WHO.
3. The authors in page 3 mentioned that "Currently, the main intervention programmes do not include PKDL control" but there are programmes for PKDL detection, diagnosis and treatment. Integrated case detection strategy is in place in leprosy hospitals, health workers have been trained in PKDL case detection and diagnosis. Further, there are guidelines for PKDL diagnosis and treatment.
4. Page 5, the authors mentioned that "the current focus of the control programme is in the so-called “endemic” administrative units, although the actual definition used for this classification is unclear". Although these are administrative units and programme unit for VL control, the target was set to have almost equal populations in those administrative units.

Does the article adequately reference differing views and opinions?

Yes

Are all factual statements correct, and are statements and arguments made adequately supported by citations?

Yes

Is the Open Letter written in accessible language?

Yes

Where applicable, are recommendations and next steps explained clearly for others to follow?

Yes

Is the rationale for the Open Letter provided in sufficient detail?

Yes

Reviewer Expertise:

Microbiology and epidemiology

I confirm that I have read this submission and believe that I have an appropriate level of expertise to confirm that it is of an acceptable scientific standard.

# Peer review: Reviewer response for version 1

**DOI:** 10.21956/gatesopenres.14209.r28147

**Published:** 2019-11-12

**Authors:** Mitali Chatterjee

**Affiliations:** 1 Department of Pharmacology, Institute of Post Graduate Medical Education and Research (IPGMER), Kolkata, India

The article has clearly highlighted the contribution/relevance of mathematical modelling in the VL elimination programme in terms of identifying the successes and limitations of the programme. 

 The authors have used the term' perverse incentive' in many places but I felt that the meaning did not come through owing to the slightly 'convoluted' sentences used to describe it. It should be elucidated in a more simple manner to enable readers to understand what they wish to convey. A few grammatical errors need to be corrected.

 I would liked to have seen a sentence or two on limitations of mathematical modelling.

Does the article adequately reference differing views and opinions?

Yes

Are all factual statements correct, and are statements and arguments made adequately supported by citations?

Yes

Is the Open Letter written in accessible language?

Partly

Where applicable, are recommendations and next steps explained clearly for others to follow?

Yes

Is the rationale for the Open Letter provided in sufficient detail?

Yes

Reviewer Expertise:

Molecular diagnostics, Immunopharmacology

I confirm that I have read this submission and believe that I have an appropriate level of expertise to confirm that it is of an acceptable scientific standard.
